# ForMileS: A Python Open-Source Program to Generate Molecular Structures for Tandem Mass Spectrometry Fragment Ions

**DOI:** 10.1021/acsomega.5c08184

**Published:** 2025-10-25

**Authors:** Vinicius Kuchenbecker, Nelson H. Morgon

**Affiliations:** Chemistry Institute, Universidade Estadual de Campina (UNICAMP), Sorocaba 18087-101, São Paulo, Brazil

## Abstract

Tandem mass spectrometry is a central analytical tool in chemistry, yet the fragmentation mechanisms underlying collision-induced dissociation remain incompletely understood. A key challenge is predicting fragment ion structures while preserving the essential structural features of the precursor ion. This paper introduces ForMileS (Formation of Mass SMILES), a streamlined Python open-source workflow for generating fragment ion structures with precursor-specific constraints from tandem mass spectrometry data. ForMileS employs a simplified branch-and-bound algorithm, accepting molecular formula, charge state, exact mass, and a base scaffold in SMILES format as input, along with parameters for branching, cyclicity, and bond types, via a graphical user interface. We demonstrate its application to the three main fragments of Polypropylene Glycol Octamer (PPG8), discussing the critical role of the base molecular scaffold (BMS) in the final structure set. Relative energy calculations using Density Functional Theory confirm the presence of expected structures, highlighting the lowest energy conformers. When applied to the smallest fragment of dipropylene glycol dimethyl ether (DGDE), ForMileS reveals that only linear double-bonded or cyclic structures are plausible, with the former being energetically favored. While successfully generating plausible structures, the exhaustive combinatorial charge generation step and the unrefined branch-and-bound method limit ForMileS’s performance, restricting its applicability to small molecules like C_6_O_3_H_19_. This highlights the importance of future performance optimization through heuristics and energetic filters.

## Graphical abstract

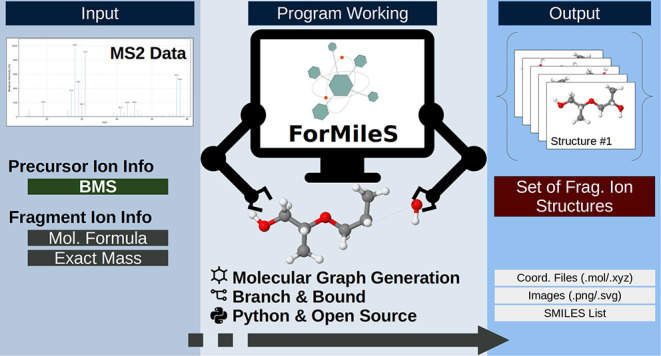

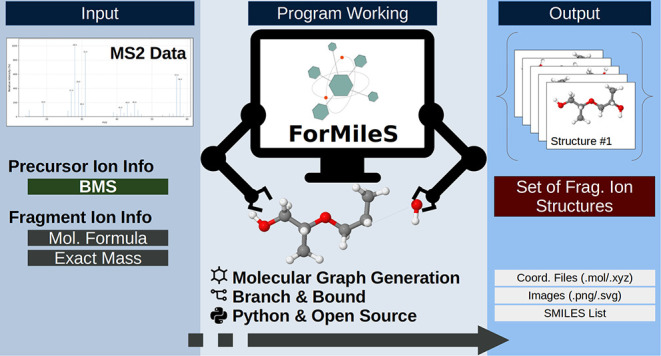

## Introduction

Among the many mass spectrometry (MS) techniques and devices, tandem mass spectrometry (MS^2^) has become a widely used method for analyzing polar and macromolecules such as polymers, carbohydrates, proteins, peptides, and others.^1^ In MS^2^ experiments, a molecular ion (also called a precursor ion) is isolated and then fragmented using various methods, the most common being collision-induced dissociation (CID).^2,3^ This process generates an ensemble of fragment ions recorded in a mass spectrum. The precursor is a positively or negatively charged molecule (ionized), with the former being more common and also referred to as a protomer. In a low energy regime dissociation process, typically one fragment retains the original charge from the precursor, while the other (or others) are neutral. In cases such as in electron transfer dissociation more charged fragments can be generated as well.^2^

Over the past years, many theoretical approaches have been developed to elucidate the dissociation mechanisms, an inherently challenging problem, and to predict and explain the fragmentation process in MS^2^, such as transition state theory^4,5^ and Rice–Ramsperger–Kassel–Marcus (RRKM) theory.^5^ All these approaches require, at least theoretically, knowledge of the precursor and fragment ion structures so that the reaction pathway (intrinsic reaction coordinate) can be modeled and energy barriers calculated.

In MS^2^ performed on most common commercial devices, such as triple quadrupoles (QqQ), quadrupole time-of-flight (qToF), and higher-energy collisional dissociation Orbitraps (HCD-Orbitrap), particularly the latter, the experimental data directly include the exact mass of the fragment ions, their charge state, and the collision energy in the laboratory frame.^3,6^ Additionally, the precursor structure can often be partly or fully known from other analytical techniques applied before the MS^2^ experiment. With exact mass measurements to four decimal places, as is common with Orbitrap analyzers, it is possible to apply algorithms for elemental composition determination, providing molecular formulas for any peak in the spectrum, including MS^2^ fragment ions.^6^

A major challenge in theoretical modeling of MS^2^ dissociation/fragmentation pathways lies in determining the molecular structures of the fragment ions—information not directly available from CID/HCD experiments. Without at least a small set of theoretically plausible fragment ion structures, attempts to model reaction mechanisms and spectra are impractical.^5,7^

One way to address this issue is through generation of all possible molecular structures for a fragment ion, i.e., a complete search for all constitutional isomers. Since the molecular formula of a fragment ion can be derived from the MS^2^ experiment, alongside other structure-related information as described above, the task becomes a combinatorial problem over the constituent atoms.^8^ Generating constitutional isomers from a molecular formula is a standard task in computer-assisted structure elucidation (CASE), which employs computational algorithms implemented in dedicated programs.^9,10^

Several programs have been developed to generate sets of molecular structures,^10–15^ notably those following a *de novo* approach, where MS^2^ fragmentation data and computational tools are used to propose precursor ion candidates, as in metabolomics applications. However, as noted earlier, in this work the focus is on supporting elucidating fragment ion structures using available structural information from the precursor ion.

Two main approaches for MS^2^ CASE are found in the literature: molecular graph generators (MGGs) and machine learning (ML) tools. ML methods use trained data sets, which greatly reduce search time but may lead to nonexhaustive solutions, as with stochastic methods,^10,16,17^ potentially missing important structures and are highly dependent on databases for training. Despite that, they are very fast and tackle the essential problem of long and exhaustive generation in some MGG algorithms. Applications such as CFM-ID,^1,18^ MetFrag,^19^ MassFrontier,^20^ and PyFragMS^21^ have been developed primarily for precursor ion elucidation from MS^2^ data, rather than for fragment ion structure generation.

MGGs tackle the combinatorial task of generating all possible structures within a framework of rules derived from experimental or theoretical data (e.g., spectra, valence, and bond order rules). This is an NP-complete problem,^8,9^ meaning that while a proposed solution can be quickly verified against the rules, finding the solution may require exponentially increasing computational effort as molecular formula size grows. Achieving both exhaustiveness and computational feasibility is therefore a significant challenge.

Existing MGG programs use various strategies and algorithms to balance completeness with computational constraints. The most common approach is structure assembly, in which molecular structures are built atom-by-atom from a given set of atoms using a defined algorithm. Examples include MOLGEN,^22^ MAYGEN,^15^ SURGE,^14^ MASS,^23^ and SMOG,^11^ with MOLGEN being considered a gold standard but available only under a commercial license.

Of these, MASS and its successor SMOG are notable for using adjacency matrices to represent molecular graphs. They are based on the branch-and-bound (B&B) algorithm of Igor Faradjev,^9^ a method dating back to the 1970s that remains important for molecular graph generation. Despite being a method that can achieve exhaustiveness with a relative simple algorithm implementation, the computational and time cost for such must be balanced in each developed program that uses B&B as base algorithm.^8^ Nonetheless, it is a method that balances simplicity and some level of exhaustiveness, depending on its application.

To manage the combinatorial explosion inherent to molecular graph generation, B&B builds structures atom-by-atom from an initial molecular scaffold, applying constraints to ensure canonicity.^24,25^ Therefore, B&B implementation and efficiency is highly dependent on the choice of constraints that will be used by the program.

While these algorithms remain effective, their implementation has traditionally relied on compiled languages such as C, Java, or Fortran—reflecting the technological context of their original development between the 1980s and 2000s. However, the growing demand for accessible, modifiable, and integrative tools in computational chemistry has prompted a shift toward more flexible environments. In this regard, Python has emerged as a leading high-level language in scientific programming,^26^ offering concise, readable syntax and a vast ecosystem of libraries. This accessibility makes it particularly suitable for chemists without extensive experience in lower-level programming.

Within Python’s extensive ecosystem, RDKit has become a key tool for computer-assisted chemistry.^27^ Its ability to read, process, and write chemical data, including structural formats such as SMILES,^25,28^ facilitates interaction between chemists and computational tools. SMILES also ensures a degree of structural canonicity, which is essential for completeness in molecular graph generation.

While existing programs can generate isomers and compare them with MS^2^ or other spectral data for graph construction or pruning,^10^ to the best of our knowledge, no B&B-based MGG written in Python has been reported that specifically combines molecular formula, charge state, exact mass, and precursor ion structural features to exhaustively generate fragment ion molecular structures for theoretical studies.

This paper introduces ForMileS (Formation of Mass SMILES), a deterministic MGG Python workflow designed to construct valid molecular graphs that are consistent with a target molecular formula for fragment ions. The program implements the established recursive B&B expansion algorithm to generate structures, commencing from a user-defined SMILES template pertinent to the precursor ion structure. The expansion process proceeds atom-by-atom, while strictly adhering to constraints such as atomic valence, bond orders, molecular formula satisfaction, and other MS^2^-related parameters, including charge state and exact mass. ForMileS subsequently outputs a collection of fragment ion structures generated in accordance with the precursor ion and MS^2^ information provided by the user.

Table 1 presented a summary of the already established programs and their method for generation of structures and main input parameters alongside ForMileS for comparison.

**Table 1:** Comparison between ForMileS and Other Molecular Graph Generator Programs Features

feature/program	ForMileS	MOLGEN	SMOG	RDKit EnumareLibrary
Input definition	Molecular formula + scaffold (SMILES), optional charge, mass target, tolerance	Molecular formula, optional constraints (e.g., connectivity, symmetry reduction)	Molecular formula, connectivity rules	Reaction definition (scaffolds + R-groups or building blocks)
Generation method	Recursive graph growth with pruning (branch and bound style) + optional scaffold seeding	Exhaustive combinatorial generation of constitutional isomers with symmetry pruning	Exhaustive canonical generation using orderly generation	Enumeration by applying substituents to predefined cores (combinatorial chemistry)
Constraints available	Branching allowed/forbidden, cyclic allowed/forbidden, max double/triple bonds, valence rules	Symmetry avoidance, max valence, atom counts, connectivity	Canonical order generation, valence rules	Library constraints defined by user (lists of R-groups)
Charge handling	Adds charges postgeneration to specific atom types	Typically neutral structures (extensions exist for charged)	Typically neutral	Charges only if defined in input scaffold
Mass filtering	Postgeneration exact mass filter with tolerance	Possible via constraints in formula	Possible via rules	Not natively; must filter externally
Output formats	SMILES, PNG/SVG, optional MOL/XYZ	SMILES, SDF, in-house formats	SMILES, SDF	SMILES, Mol objects (in-memory)
User interface	GUI built in Tkinter + Command Line Interface (CLI)	CLI only (commercial academic license)	CLI	Python API (scriptable)
Unique aspect	Formula + mass tolerance targeting (for MS); Scaffold-seeded growth; Charge placement automation; Graph expansion	Very mature, optimized, symmetry exact and exhaustive	Compact canonical representation	Tight RDKit integration, combinatorial enumeration

The program is not intended to replace existing tools (which are better at exhaustive and efficient generation) but rather to complement them, representing a modern and specific workflow to perform MGG specifically in the MS^2^ context. The main contribution of ForMileS is the integration of the aforementioned MS^2^ specific constraints with the B&B algorithm. Indeed, as will further be demonstrated in the next sections, ForMileS has aspects of both a generator and confirmation tool for molecular structures.

Hence, ForMileS is introduced as a support tool, positioned within a specialized area between exhaustive MGG methods, which are less MS^2^ aware, and programs dedicated to the structural elucidation of precursor-focused MS^2^.

## Implementation and Computational Method

### Description of Program Functionalities and Algorithm

Figure 1 summarizes the complete ForMileS workflow in pseudocode form, highlighting how each numbered block corresponds to a functional module later described in the text. Table 2 expands these modules, translating every pseudocode block into its explicit Python implementation and indicating the main functions or objects invoked in the source code. For example, the pseudocode segment “GrowRecursive­() → ProcessCompleteMolecule­()” (Figure 1, lines 6–9) is detailed in Table 2 under “Graph generation,” where the recursive growth, valence validation, and pruning criteria are explained through the functions grow_recursive and valence_ok. Likewise, the pseudocode step “GenerateChargedSMILES­() → FilterByMass­()” is linked to the “Charge generation” and “Filtering” rows of Table 2 and elaborated in the following subsections. This cross-reference aims to help to trace the execution flow from algorithmic logic (Figure 1) to implementation (Table 2) and explanatory discussion.

**Figure 1:**
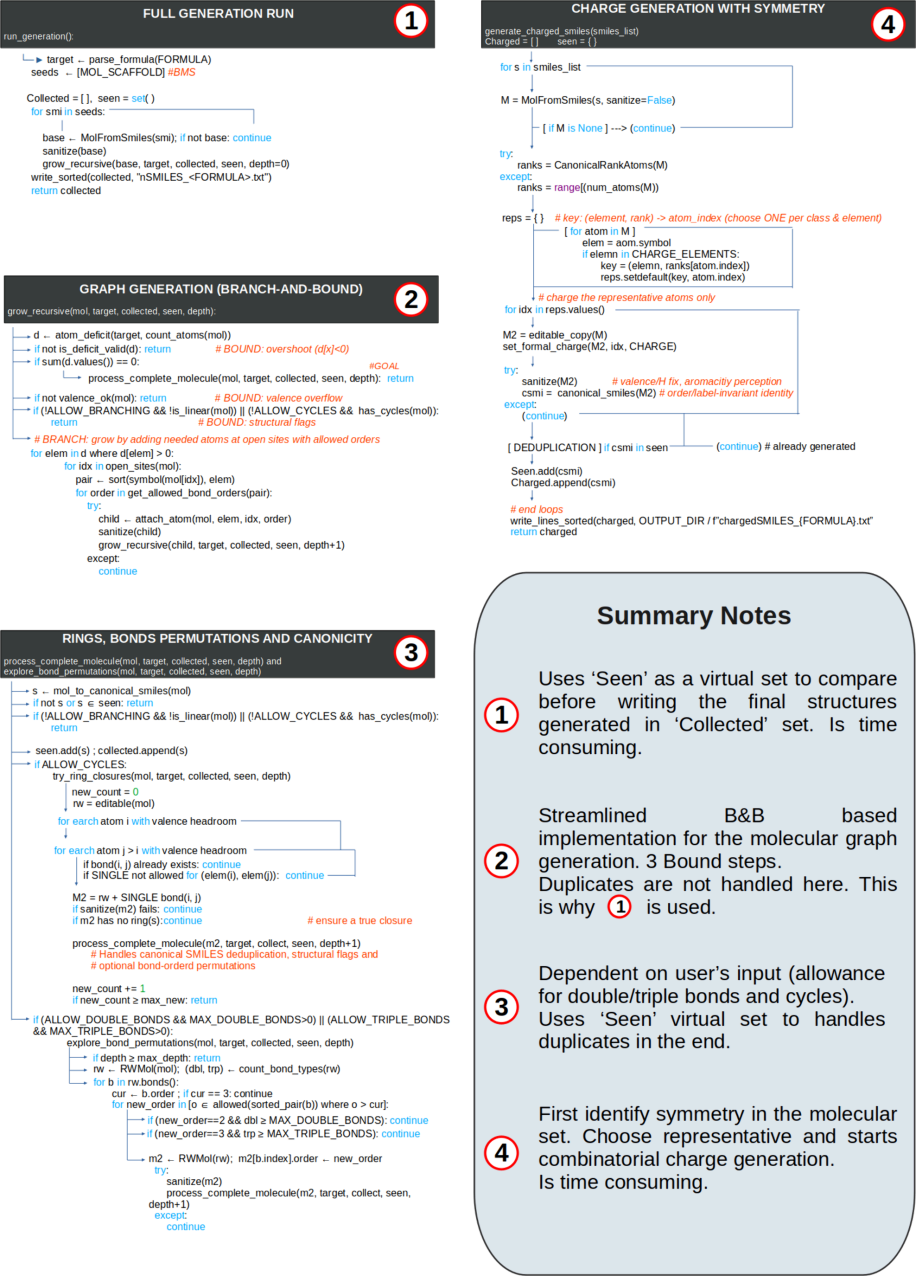
ForMileS simplified workflow with most important molecular graph generation functionalities explained using pseudocode of Python’s script.

**Table 2:** Description of ForMileS Main Functionalities and Their Correspondence to the Implemented Script Elements

program step	description	script elements
Graph generation	Parses target formula, then recursively grows from each scaffold: adds atoms to “open sites” only if (i) valence limits per element, (ii) allowed bond orders by element pair and global flags, (iii) structural constraints hold. When the atom deficit hits zero, the structure is considered complete.	parse_formula, open_sites, get_allowed_bond_orders, valence_ok, grow_recursive, process_complete_molecule
Ring handling	If rings are allowed, tries ring closures by adding bonds between atoms that still have valence headroom; only keeps sanitizable ringed structures.	try_ring_closures (called inside process_complete_molecule)
Bond-order permutations	For completed molecules, optionally explores higher bond orders (double/triple) bounded by global maxima to avoid combinatorial blow-up.	explore_bond_permutations (invoked from process_complete_molecule)
Duplicate control (neutral)	Every completed structure is converted to canonical SMILES; a seen set blocks duplicates immediately. Structural constraints are checked again at this boundary.	mol_to_canonical_smiles, process_complete_molecule (with seen), has_cycles, is_linear
Charge generation	For each neutral SMILES, uses RDKit’s symmetry ranks to choose exactly one representative atom per symmetry class for the allowed chargeable elements and applies the formal charge. After sanitization, canonical SMILES ensures dedup.	generate_charged_smiles (uses Chem.CanonicalRankAtoms, SetFormalCharge, Chem.MolToSmiles)
Filtering	Exact mass filter (monoisotopic) against TARGET_MASS ± TOLERANCE, with canonicalization-based dedup for the filtered set.	filter_by_mass using Descriptors.ExactMolWt
Artifacts	2D annotated PNGs (and optional SVG), optional XYZ/MOL via 3D embed + UFF optimize, plus a human-readable run summary with timings, RAM snapshots, and final counts.	smiles_to_images, generate_xyz_from_smiles, generate_mol_from_smiles, _write_run_summary
Performance snapshots	Lightweight RAM probe (psutil if available; resource fallback), timing with perf_counter().	_get_ram_mb, main __name__ = = ″__main__″ block

### Molecular Graph Generation

While ForMileS does not contribute to the B&B algorithm itself, it implements core elements of it to graph generation within the aforementioned workflow to recursively grow the molecular graph atom by atom.^9,11^ This process can be represented as a decision tree, where each level corresponds to the addition of one atom beyond the BMS atoms defined as input. The nodes represent molecular graphs, and the branches represent all possible ways of adding the next atom to valid sites with valid bonds. The search space is reduced through pruning (bounding). Details are found in Figure 1.

The program identifies the atoms and their quantity in the molecular formula parsed by the user, storing it in a vector $\overset{⃗}{f_{target}\;}$ . This is done using the functions parse_formula­().

Then, from the BMS, the function count_atoms­() counts all the already existent atoms in the current molecular graph *G*, storing in the vector *f⃗*(*G*). From this values, in each recursive growth of the molecular graph, i.e. each atom-adding step, the vector $\mathrm{d⃗}=\overset{⃗}{f_{target}\;}-\mathrm{f⃗}\left(G\right)$ is calculated with the function atom_deficit­() to evaluate the atom deficiency in the current graph.

The first bound condition is when *d⃗* = 0. This conditioning alone would still let the combinatory space to grow prohibitively the bigger the molecule, but satisfy the most important goal of any MGG: generating a structure that fulfills the required molecular formula.

Another important chemical constraint used for bounding is the valence of each atom. Each atom *a* have a current valence *V*(*a*) in each step of the molecular graph growing process. Using valence defined in parameters.json file, there is a *V*_max_ for each atom type, e.g. carbon has 4, oxygen 2 and etc. Because of this, a valence constraint to bound, i.e. pruning branches is that ∀*a* ∈ *G*, *V*(*a*) ≤ *V*_max_(*a*). This also means that the program is limited to work only with the explicit valence information available at the parameters file and will use ‘if not valence_ok­(current_mol, max_valence): return’ to check the valence.

Also, if any element *x* in the recursive branching tree has *d⃗*[*x*] < 0, this means the program overshot the formula and the branch is pruned. This is tested by the function is_deficit_valid­(), being deficit = atom_deficit­(target_formula, current_atoms). The process of growing branches continues for each atom, which means the program iterates over atoms still needed but skip the exhausted ones.

The program utilizes parameters.json to define valences and bond orders for atom additions. It employs recursive calls to attach_atom and grow_recursive functions for molecular graph construction, with the RWMol class facilitating molecule editing and candidate generation.

Upon valid molecule creation, postprocessing involves bond permutations for double/triple bonds and ring generation if permitted. To reduce combinatorial complexity, the user specifies the maximum number of allowed double and triple bonds.

Canonicity is not enforced by the branch-and-bound (B&B) growth. Instead, RDKit canonical SMILES ensures uniqueness, with virtual sets (called ‘seen’) eliminating duplicates at two stages: (i) neutral graphs from the B&B step, and (ii) charged variants from charge placement. This design collapses structures reachable via different growth orders or bond-order permutations into a single canonical representative.

At each B&B goal state (i.e., when atom-deficit is zero and constraints are met), the candidate graph is sanitized and converted to canonical SMILES before being admitted to the neutral pool. After charge localization, each charged candidate undergoes recanonicalization and deduplication.

### Ring System Generation

If cycles are allowed by the user, when the formula is satisfied, the algorithm may attempt ring closures by adding bonds between atoms that still have valence headroom and are topologically eligible (subject to global constraints). Double/triple bond permutations are explored within user-specified maxima to capture alternative valence patterns relevant to the chemistry. Invalid proposals (valence overflow, impossible ring strain under the simple valence model, or rule violations) are rejected immediately by sanitize/validation.

Aromaticity is not hard-coded by ForMileS. Instead, it is perceived by RDKit during sanitize­(), i.e., “kekulization”. The program grows graphs in a bond-order form. RDKit then analyzes cycles and π-electron patterns to mark atoms/bonds as aromatic when appropriate. Because canonical SMILES respect RDKit’s aromaticity perception, resonance-equivalent representations (e.g., alternating double-bond patterns in a ring) are collapsed to a single canonical form. This prevents duplicates arising from different but equivalent kekulé assignments. If the user limits the total number of double/triple bonds, that indirectly constrains which ring systems can become aromatic (some candidates will sanitize as nonaromatic if the π budget is insufficient).

Therefore, aromaticity is emergent from RDKit’s perception of the sanitized candidate. ForMileS does not enumerate “aromatic forms” separately. Canonical SMILES collapses resonance/kekulé variants, so aromatic duplicates are not produced.

Also, the cyclization process can explode in combinations, and therefore the program starts with a 10 maximum structure limitation in its script that can be changed by the user. Therefore, the cycles building process is not exhaustive as in other established MGG and would benefit from future implementations of chemical constraints.

### Charge Generation and Exact Mass Filter

Charge generation initiates with neutral molecular graphs from the B&B step. This combinatorial process, while computationally intensive, is essential as multiple sites can bear charge in an MS^2^ ion.

The parameters.json file specifies atoms capable of bearing charge. The generate_charged_smiles­() function utilizes this list with SMILES strings from the B&B step. Its primary purpose is to combinatorially assign a charge to a labeled atom, store the resulting structure, and avoid duplicates using RDKit’s symmetry check. SMILES are crucial because RDKit’s Chem.MolFromSmiles­() implicitly assigns hydrogens to heavy atoms.

For experimental data-based filtering, exact mass is employed. The filter_by_mass­() function uses RDKit’s Descriptors.ExactMolWt­() to determine if each charged SMILES falls within a specified mass tolerance, returning those that meet the criteria.

Although hydrogens are not initially assigned, RDKit functions explicitly account for them during mass calculation. Since charges are assigned prior to this step, the program can ″sanitize″ molecules by adjusting hydrogens when testing charged sites and their valences.

### The Base Molecular Scaffold

Certain programs utilize Nuclear Magnetic Resonance (NMR), Infrared Spectroscopy (IR), or MS data files as inputs for molecular graph generation, providing an initial framework to reduce combinatorial space and ensure physical feasibility. However, this approach necessitates the specific data file, thereby restricting the program’s utility to empirical or database sources. ForMileS, conversely, allows the user to provide a SMILES string as the Base Molecular Scaffold (BMS)—the initial substructure from which all conceivable molecular graphs are constructed. The BMS represents an indispensable molecular substructure of the precursor ion that must be conserved within the fragment ion structure. This is more than a mere constraint; it embodies a structural characteristic of the precursor ion that is maintained in the fragment ion.

In MS^2^ experiments, as in numerous other MS applications, chemical expertise remains indispensable. The utilization of SMILES as the BMS expands possibilities, particularly for theoretical investigations, as expounded in the Introduction. The same rationale supporting this approach can also be invoked to critique it: significant lacunae persist in our comprehension of gas-phase dissociation and fragmentation mechanisms. Consequently, the selection of the BMS is a nontrivial decision and serves as the primary determinant of the final results. A dedicated subsection in the Results and Discussion section elucidates the effects of the BMS and offers guidance on its appropriate selection.

### Technical Stack

As previously noted in the Introduction, the selection of Python as the programming language enhances script readability, minimizes code length, and facilitates seamless integration into larger computational pipelines. This choice also promotes the program’s adoption within the mass spectrometry (MS) domain, enabling experimental chemists with limited computer science expertise to comprehend the program’s underlying logic and even propose enhancements or modifications.

A crucial distinction of ForMileS from other MS^2^-context programs is its reliance on exact mass, thereby eliminating the necessity of accounting for hydrogen atoms during the combinatorial B&B steps. During the combinatorial step for generating charges, RDKit exclusively manages hydrogen atoms pertinent to the charge, such as those present in protonated alcohols. This process is streamlined by RDKit’s efficient handling of SMILES notation, which obviates the need for explicit hydrogens. Consequently, stereoisomers are not generated.

The program refrains from employing virtual fragmentation reaction techniques or data sets to assess the feasibility of dissociation products, unlike MOLGEN-MS. This approach stems from ForMileS’s design objective: to generate fundamental structures for the study of these reactions, rather than to predetermine them. While the application of reaction rules could reduce computational time, it would impose a level of reactional constraint antithetical to the program’s purpose.

### Inputs and Outputs

In contrast to programs such as MASS/SMOG, SURGE, or even MOLGEN, ForMileS produces.xyz coordinate files—one of the most prevalent formats in computational chemistry—in addition to.mol files. Although MOLGEN does generate.mol files, it is a commercially licensed program. All coordinate files are generated using RDKit’s integrated 2D-to-3D functions and necessitate thorough inspection and subsequent submission to a geometry optimization routine, which can be performed using classical force-field molecular dynamics (FFMD), semiempirical methods, or density functional theory (DFT).^29^

Finally, standard RDKit functions save images and coordinate files for the filtered SMILES list. The program also saves intermediate SMILES lists for B&B generation, charge assignment, and final exact mass filtering in the output directory.

Beyond rudimentary valence rules for atomic bonding, the absence of any mechanistic reaction reasoning in the molecular structure generation, unlike MOLGEN-MS, implies that the final result set comprises purely combinatorial and mathematically possible constitutional isomers. These isomers require the assignment of physical meaning, typically through energy calculations such as Single Point Energy, Enthalpy, or Gibbs energy. Therefore, while the lack of further constraints permits a degree of freedom in generation, it is imperative that the results undergo validation, as the program alone does not furnish chemical and physical feasibility validation for the structures and may produce chemically unsound entities, including unstable heterocycles, antiaromatic systems, and highly strained molecules.

All program parameters are input via a Graphical User Interface (GUI) and stored in a discrete parameters.json file, affording users the flexibility to experiment with varying rules for elemental orders, maximum valences, and elements capable of bearing charges. This design reflects an open-source philosophy and extends the program’s applicability across a broad spectrum of systems. Currently, the program supports commonly encountered organic elements: Li, Be, B, C, N, O, F, Cl, Br, I, Si, P, and S, with a primary focus on C, O, and N during the publication period. As testing progresses, additional elements can be incorporated.

The input parameters in the GUI are molecular formula, BMS, exact mass, enabling/disabling doubles and/or triple bonds, cycle and ramified generation and how much double/triple bonds if any allowed. The user also defines if.mol, .xyz and.svg files will be generated and the path to the output folder.

The entirety of the program is operated through a Graphical User Interface (GUI) constructed with the native Tkinter package, ensuring compatibility and mitigating unnecessary conflicts. While this interface is ubiquitous and lightweight, its visual presentation is considered outdated, and its proper resolution is highly dependent on the personal computer’s graphical configurations.

### Validation and Limitations

Given that the initial inspiration for the program’s development stemmed from the structural analysis of small ether-glycol MS^2^ fragment ions, two representative examples from this class have been selected to demonstrate ForMileS’s capabilities. From the extensive collection of glycol Electrospray Ionization Tandem Mass Spectrometry (ESI-MS^2^) data accessible in databases such as mzCloud, Polypropylene Glycol Octamer (PPG8),^35^ a polycondensation polymer, was chosen to illustrate and discuss the rationale behind BMS selection and its chemical significance, owing to its well-characterized MS^2^ profile and readily available data. Dipropylene glycol dimethyl ether (DGDE)^36^ was also selected due to its small size yet structural similarity to PPG8, serving to further exemplify the retrieval of chemical information from the results. Both examples are additionally utilized to evaluate the program’s performance and limitations. A set of small molecules reported in MAYGEN is used as well to perform benchmarking.

To illustrate the ForMileS workflow and execute theoretical energy calculations, the generated fragment structures were subjected to semiempirical geometry optimization, succeeded by a DFT frequency calculation to ascertain their relative Gibbs energy profiles. All computations were conducted using ORCA^30^ v.6.0. Geometry optimization employed the GFN2-xTB^31^ semiempirical method. Frequency calculations utilized the B3LYP^32,33^ functional with the Alrich def2-SVP basis set,^34^ and outcomes were graphically represented with the lowest energy normalized to zero for the purpose of relative energy assessment.

## Results and Discussion

### Performance Benchmarking

Table 3 compares the wall-time for graph generation in ForMileS with established MGGs: MOLGEN, MAYGEN (an open-source MMG), and OMG.^37^ All ForMileS tests were performed on an AMD Ryzen 3600 (8 cores, 16 GB RAM). Data for other MGGs were retrieved from MAYGEN’s original publication,^38^ not generated on the same setup. This comparison provides a raw view of performance.

**Table 3:** Wall-Time Comparison for ForMiles and Other Molecular Graph Generators Available

		wall-time (s)			
molecular formula	# structures	MOLGEN	MAYGEN	OMG	ForMileS
C_3_Cl_2_H_4_	7	0.006	0.070	0.219	0.049
C_3_O_3_H_4_	152	0.026	0.110	0.389	1.462
C_6_H_6_	217	0.049	0.159	0.454	1.153
Cl_2_C_5_H_4_	217	0.028	0.116	0.659	4.930
C_5_H_9_ClO	334	0.009	0.147	0.745	16.268
C_6_OF_2_H_12_	536	0.032	0.210	6.219	2385.403

For this comparative assessment, ForMileS was executed without any constraints. Double and triple bonds were permitted to be generated indefinitely, as were ramified and cyclic structures, and no base scaffold (BMS) was provided. This is reflected in the fact that all programs generated precisely the same number of structures. The sole constraint is the inherent script of the program, which does not explicitly utilize hydrogens in the combinatorial B&B phase. Furthermore, no charge was added, as the data set employed consists of neutral molecules.

It is evident that ForMileS exhibits poor scalability when compared with established MGG methods, reinforcing that it is not an exhaustive generator suitable for replacing existing ones. While many larger molecular formulas are available for benchmarking, the extensive generation time observed for the last molecular formula already clearly indicated the program’s significant limitation in achieving efficiency without constraints.

For the smallest molecule (C_3_Cl_2_H_4_) alone, ForMileS outperformed MAYGEN and OMG. This underscores the fundamental limitation of the B&B algorithm with respect to larger systems.^9^ Moreover, its current implementation is simplified. For truly efficient graph generation, we endorse the preeminent positions of MOLGEN and even MAYGEN as graph generators. These programs also possess their own ’good’ and ’bad’ lists that support the program in generating structures, and, as anticipated, no naive B&B implementation can approximate such efficiency. Despite this, exhaustiveness for small molecules was achieved, satisfying one of the primary concerns regarding ForMileS’s performance.

Therefore, the streamlined B&B implementation necessitates future revisions and algorithmic updates to achieve comparable efficiency with standard tools, and we present this version of the program as the foundational step toward that goal.

The computational scaling limitation highlights the importance of evaluating the influence of the BMS parameter on both time, memory, and, critically, the final result set. Figure 2 summarizes the impact of BMS selection for the three main fragments of PPG8 concerning wall-time and the quantity of structures generated.

**Figure 2:**
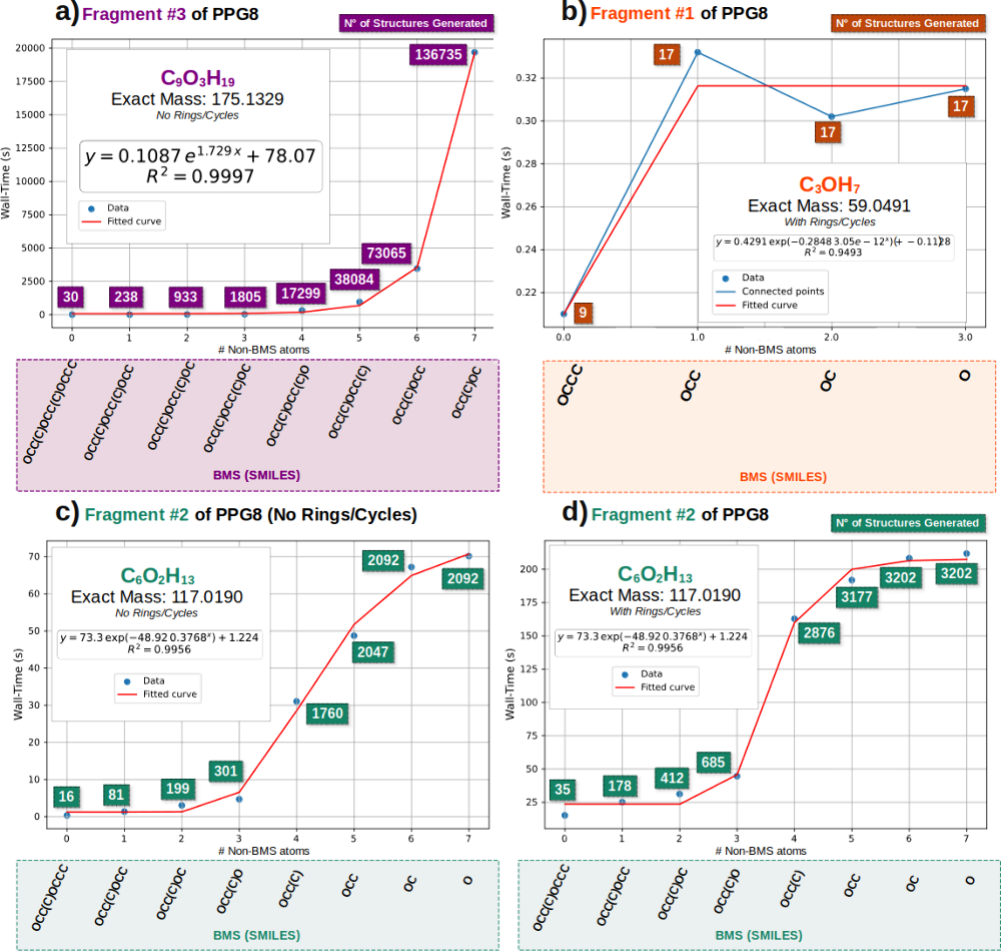
Wall-time as a function of number of nonscaffold atoms with SMILES used as BMS and total of generated structures for a) Fragment #3, b) Fragment #1, c) Fragment #2 without rings generated, d) Fragment #2 with full generation, including rings.

For all generation tests performed with PPG8 fragments, ramified structures were permitted, along with an extensive allowance for double and triple bonds. The primary parameter investigated in conjunction with BMS was the generation of ring structures, as detailed below.

The initial and most crucial observation is that BMS significantly impacts wall-time as the system size increases. With Fragments 1 and 2, the computational resources facilitated a comprehensive investigation into the effect of the number of non-BMS atoms—ranging from no B&B growth (number of non-BMS atoms maximized) to purely combinatorial, without BMS (which is represented as only one atom input). As Fragment 1 (Figure 2b) is small, its behavior is less distinct for validation, given that only four BMS were permitted. However, it is evident that with just one fewer atom in the BMS compared to the purely combinatorial no-BMS, the wall-time fluctuates around a mean value. Regarding the number of structures, the quantity remains constant whether ’OCC’ or ’O’ is employed, indicating convergence for this exceptionally small molecule.

It is with Fragment 2 (Figure 2c and d) that the anticipated behavior is more clearly discernible. Gompertz logistic regression aids in visualizing a substantially reduced (though significant) change in the wall-time of generated structures within the region of pure combinatorial generation (No BMS) and complete BMS. In the middle of the spectrum, the simple decision of adding or removing an atom from BMS can lead to astonishing changes in wall-time and in the number of final structures. This is also contingent on the size of the molecular formula, as observed when comparing Fragment 2 with Fragment 3. While the graphical representation may be misleading, even a transition from a full BMS to 1 fewer atom in Fragment 2 and 3 resulted in 80% and 87% more structures generated, respectively. For Fragment 3, a difference of 6 to 7 atoms in the BMS led to an 85% increase in wall-time, while yielding a 46% increase in the number of structures generated in the final set.

In the case of Fragment 3 (Figure 2a), for a molecule of this size, generation ceased when the number of non-BMS atoms reached 7, due to time limitations, as this step required almost 5 h and 30 min of generation. Should the process continue to the limit of no-BMS, a convergence behavior would be expected, but it would most likely be prohibitive in the time domain. Furthermore, to further mitigate time consumption for profile generation, no ring structures were permitted for Fragment 3. From the tests with Fragment 2, it is evident that cycle generation (Figure 2c,d) drastically increases time consumption but does not alter the overall behavior (shape of the curve); the same is assumed for larger systems. At the limit of full generation, allowing rings generated approximately 35% more structures at the expense of 65% more time. Despite this, in Fragment 2, both with and without ring generation, the last two BMS entries yielded the same number of structures, indicating convergence.

Another significant source of computational cost and delay is the fully combinatorial charge generation step. Figure 3 presents a comparison of the actual B&B graph generation and charge generation steps for the aforementioned Fragment #2 of PPG8, along with an overall summary of Linear versus Cyclic generation.

**Figure 3:**
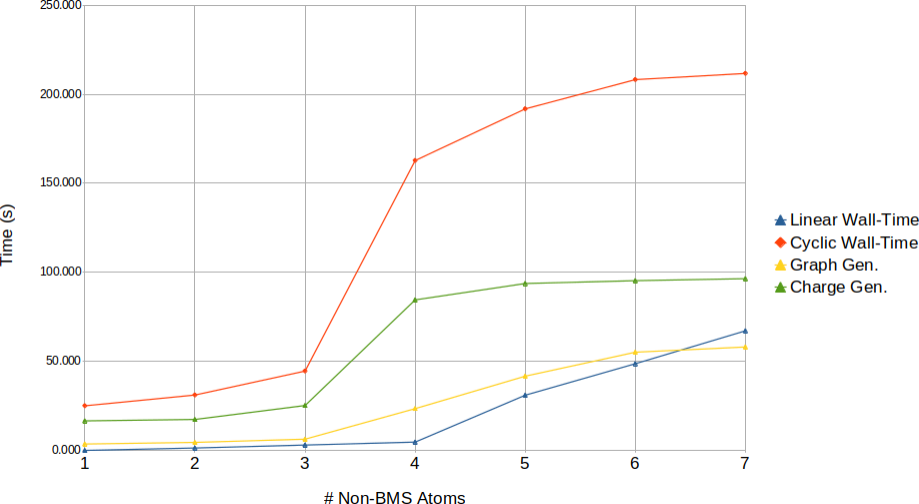
Overlaid comparison of time for Fragment #2 generation with/without cycles formation just charge and just graph generation steps of ForMileS.

As seen in Figure 3, for larger BMS, the gap between graph and charge generation narrows, with charge generation becoming the primary bottleneck. This phase, being O­(n^k^) with k charges across n sites, significantly impacts ForMileS’s performance. The exhaustive nature of charge generation, which considers every atom specified in parameters.json for charge assignment, currently dictates this cost. While exhaustive, future heuristic rules could potentially improve performance without compromising chemical conclusions.

Figure 4 illustrates using Fragment 2 and 3 that memory consumption follows the same growth trend as wall-time.

**Figure 4:**
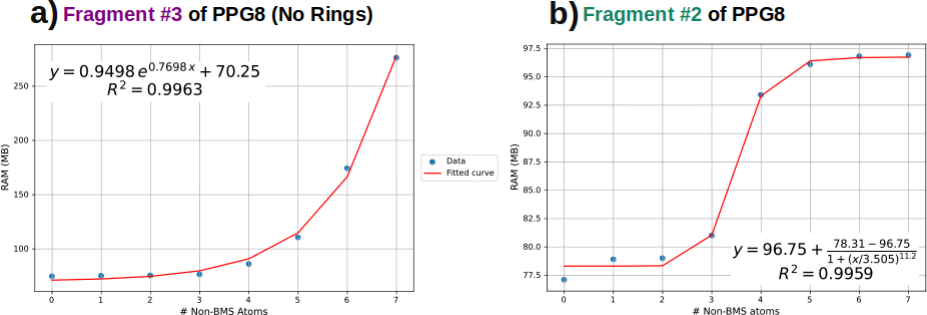
RAM memory as a function of number of non-BMS atoms in ForMileS run for a) PPG8 fragment #3 without cycles allowed, b) PPG8 fragment #2 with cycles allowed.

These findings indicate that the ultimate quantity of generated structures and the time expended are predominantly contingent upon the selection of the BMS although other parameters, such as the allowance for ring formation, do exert a significant influence in wall-time as well. While this can significantly reduce computational time, it concurrently diminishes the final molecular set.

This outcome is incongruous if a new Molecular Graph Generator (MGG) designed for efficient generation of a comprehensive molecular space is intended. However, given the promise of a tool specifically tailored for the MS^2^ context, ForMiles can be used with the BMS judiciously chosen based on a set of criteria to generate structures suitable for dissociation studies. Nevertheless, based on these initial performance results, ForMileS is only recommended for relatively small molecules. Currently, the largest molecules tested without constraints are C_6_OF_2_H_12_ or C_9_O_3_H_19_, with a BMS constraint of 5 atoms, and even these necessitate several minutes for generation.

The ensuing section will elucidate and deliberate upon the chemical validity of the generated structures, alongside providing guidance for BMS selection to ensure an acceptable set of structures within a viable computational time frame.

### The Role of Base Molecular Scaffold

Based on the previously discussed effect of BMS in the final number of structures generated, this is the main parameter to be evaluated when using ForMileS. The initial steps in determining the BMS involve: (i) ascertaining the molecular formula of the fragment and precursor ion structure; (ii) identifying potential precursor cleavage sites that yield a fragment with the experimentally observed exact mass; and (iii) recognizing consistent atom connectivity among all dissociation groups. For polymers, if the fragment’s exact mass corresponds to a multiple of the monomer unit, this is typically a precursor feature to be preserved during fragmentation. Finally, (iv) common structural features retained across all hypothetical precursor cleavages, as depicted in the gray dotted area of Figure 5a, represent the primary BMS candidate.

**Figure 5:**
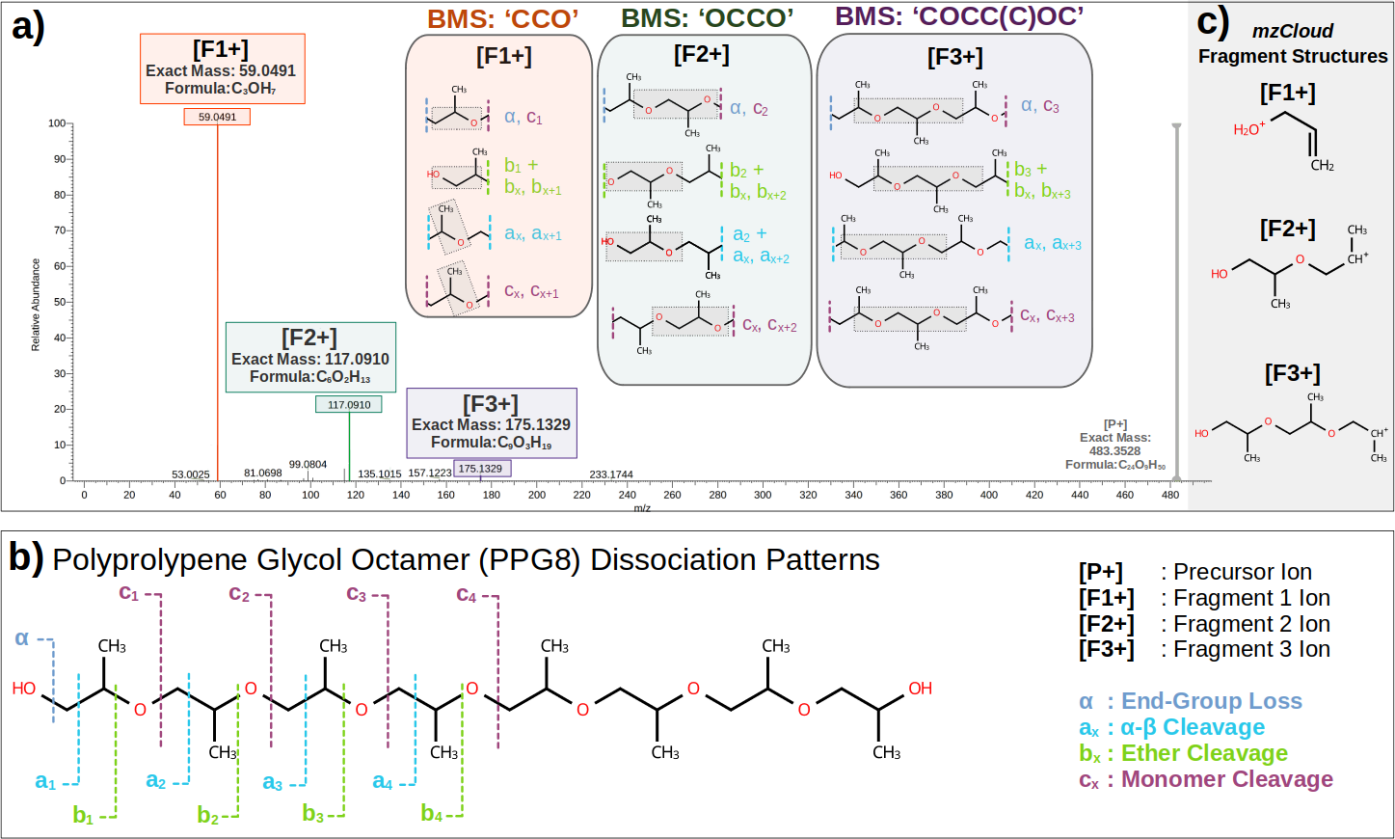
a) ESI-MS^2^ spectrum for PPG8 from mzCloud database.^35^ BMS for ForMileS are highlighted with the dissociation patterns that led to them. mzCloud fragment structure is in a gray shaded area. b) PPG8 dissociation patterns and cleave site labeling. Adapted with permission from ref 38. Copyright 2011 Wiley & Sons.

Desirable BMS characteristics include: a) maximal distance from the anticipated bond fragmentation region during dissociation; and b) minimal reactivity with respect to functional group populations. These considerations are pertinent to typical CID/HCD processes occurring in the tens of μs to ms time scale.^3^ While various rearrangements can occur, some may be time-limited before the fragment reaches the analyzer. A larger fragment and a BMS more distant from the fragmentation site reduce the likelihood—though do not eliminate it—of the proposed BMS not being a preserved molecular precursor feature. A more reactive chosen BMS increases the probability of it not being a nontransforming structural feature of the precursor, thus potentially excluding important fragment structures.

This ″cut-and-recognize patterns″ process is complex across different molecules. Depending on the precursor and fragment type and size, knowledge of common neutral losses can be beneficial.

Regardless of the aforementioned guidelines, the BMS is a hypothetical choice that significantly biases the final result. Its sole commitments are to the fragment’s exact mass and the assumption that the chosen substructure is genuinely retained. The flexibility to test comes with the cost of bias, obliging the user to gather extensive data and theoretical information for BMS selection, acknowledging that it is a conjecture requiring further evaluation. Common neutral losses, as detailed in Tandem Mass Spectrometry literature, serve as valuable starting points.

### Case Study 1: PPG8 Fragment Analysis

Figure 5a presents the ESI-MS^2^ spectrum available at mzCloud. This is reported as a Higher-Collision Energy Dissociation (HCD) process with 40% Collision Energy. The three main fragments are presented in order of relative intensities. In the shaded area in the right corner (Figure 5c) there are each of the reported molecular structures for the fragments by the mzCloud database system.

Previous works^39–41^ have systematically studied the pattern of bond dissociation in polyethers and polyesters. Figure 5b illustrates a schematic division of potential cleavage sites in PPG8, revealing a repetitive pattern despite the molecule’s size.

In the case of PPG8, for any given fragment, it is possible to exhaust the breakage patterns that would yield the exact fragment mass in accordance with experimental data. For instance, ’α+b_*x*_’ cannot generate any fragments with an exact mass for this particular dissociative process, though it is not chemically prohibitive.

Indeed, this pattern does not represent a reaction dissociation step or mechanism; rather, it serves as a map of hypothetical cleavage sites within the precursor that would generate fragments fitting the observed exact mass. The gray dotted area in Figure 5a for each fragment box signifies the common structural section found in all possible cuts made in precursor ions to generate hypothetical fragments. This is not yet a chemical proposition of a reaction mechanism, but rather a structural hypothesis that currently satisfies exact mass and precursor structural features.^39^

The common protonated PPG8 form in ESI-MS^2^ is the end-group R–OH_2_^+^, which readily loses H_2_O in CID/HCD.^3,6,39^ Therefore, in the ‘α, C_*x*_’ cleavage proposed in Figure 5a, end-group fragmentation is anticipated. This suggests that three carbons connected with an end oxygen group are more probable than a ‘CCOC,’ despite the latter’s potential generation via an ‘a_*x*_ to a_*x*+1_’ process. Between ‘CCO’ and ‘COC,’ the former appears more reasonable or general, allowing for both ether and alcohol structures for this fragment’s molecular formula, whereas ‘COC’ limits the final set to molecules containing only ether functional groups. Although ether is thermodynamically more stable, for very small fragments, numerous rearrangements can occur.^3,5,6^ Thus, for larger, more complex molecules, reactivity considerations are crucial. However, for this small fragment, B&B performs well, and fewer constraints, such as no BMS, can be applied. Despite this, Figure 3b shows that both no BMS and ‘CCO’ yield the same converged result. Consequently, if time permits, testing different BMS options can evaluate their impact on computational time and the number of generated structures.

As is commonly applied in many cases of molecular structure set evaluation, geometry optimization followed by a frequency calculation—or even simple Single Point Energy calculations—can be employed to assess the energetic profile of the resultant outcome and facilitate the evaluation of physically and chemically valid structures while excluding those with excessively high energy.^4^

To exemplify this methodology, we selected the most spectrally intense fragment set, F1+, and subjected it to semiempirical geometry optimization using a robust and dependable semiempirical method, GFN2-xTB,^31,42^ followed by a straightforward DFT/B3LYP/def2-SVP^32,33^ frequency calculation to determine Gibbs energy. Given that the objective is to ascertain relative energy differences between structures expected to exhibit significant variations, the B3LYP functional with the single-ζ Alrich basis set was chosen. Large systems may also substantially benefit from the incorporation of D3 dispersion corrections.^43^

To aid in the validation of the ForMileS output set, we investigated the presence of the fragment structure proposed by mzCloud itself (Figure 5c). It was anticipated that at least one of the generated structures would correspond to the published fragment. Furthermore, the Gibbs energy calculation was utilized to identify the most stable structures in the gas phase. The results are summarized in Figure 6.

**Figure 6:**
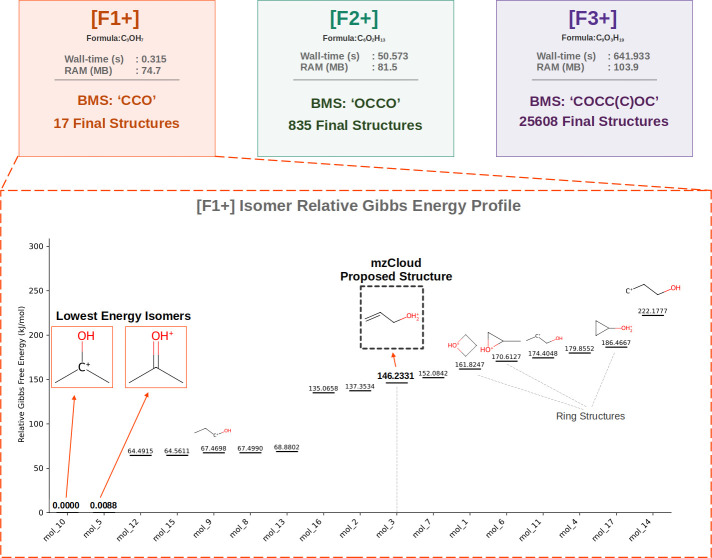
Relative Gibbs energy profile for structures generated for Fragment #1 of PPG8. Lowest energy structures are highlighted in orange.

The presence of mzCloud molecular structures for F1+ within the generated set is consistent with the appropriate selection of BMS. This observation is similarly noted for F2+ and F3+, thereby contributing to the validation of the ForMileS output.

Furthermore, two generated structures are highlighted as the lowest energy isomers within the set. While frequency calculations are sensitive to the method and basis set for accurate energy level determination, the objective herein is to isolate structures within a defined relative energy range.^42^ It is observed that these two structures exhibit an energy difference exceeding 60 kJ/mol from the subsequent low-energy isomers. Notably, the mzCloud allyl-protonated alcohol structure presents an energy level greater than 146 kJ/mol higher when compared with the secondary protonated carbonyl and the secondary carbocation adjacent to the alcohol.

Additional generated structures, such as the highly unstable primary linear carbocation (which represents the highest energy structure) and the unique ring structures (positioned toward the high-energy end, particularly due to ring strain in 3- and 4-membered rings), are depicted in Figure 6.

It is well-established that tertiary carbocations exhibit greater stability than secondary and primary carbocations, particularly in linear molecules and in the absence of electron-donating groups such as heteroatom oxygen. Although a more comprehensive discussion on this topic is feasible, it falls outside the scope of the current work.,^4,44^ ^45^

The presented workflow, while straightforward, is essential for imparting chemical meaning with a reasonable degree of reliability to the ForMileS output set. Users are strongly encouraged to employ diverse computational chemistry methods and analytical techniques to verify the output. This aligns with the program’s capability to generate coordinate files.

### Case Study 2: DGDE Fragment #1 Analysis

Figure 7a shows the spectrum for DGDE as reported in the mzCloud database. The spectrum was recorded using a relative HCD (High energy Collision Induced Dissociation) energy of 40% in a HCD cell. For this molecule, mzCloud does not provide a molecular structure for each fragment, just the MS^2^ data.

**Figure 7:**
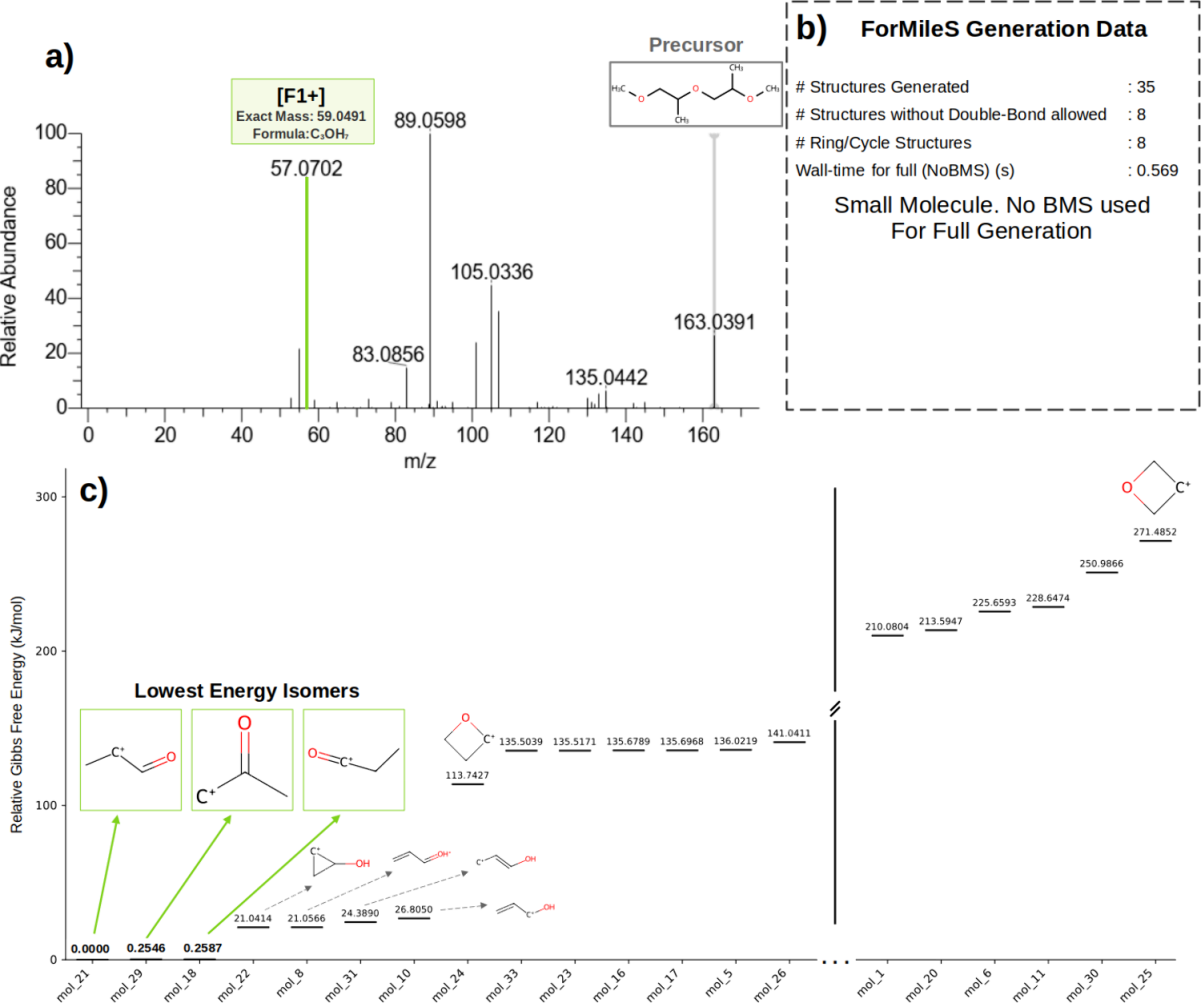
a) ESI-MS^2^ spectrum for DGDE b) data related to ForMileS execution for Fragment F1+ of DGDE c) Relative Gibbs energy for generated structures with the lowest in energy highlighted in green box.

This molecule possesses structural elements analogous to polypropylene glycol dimer, featuring two methyl ether end-groups rather than hydroxyl. The hypothetical BMS selection could align with the previously discussed principles. Nevertheless, as previously demonstrated and elaborated, for a molecule of this magnitude, the program can be utilized without any BMS and still successfully generate all structures within a reasonable time frame.

For F1+, particularly, significant information can be gleaned from the final output set generated with cycles included. As depicted in Figure 7c, only double-bonded or cyclic structures satisfy the exact mass, even in the absence of a BMS. Disallowing double bonds leads ForMileS to generate solely cyclic structures. However, as also presented, the cyclic structures exhibit an energy level at least 21 kJ/mol higher than the double-bonded structures, which represent the lowest energy in the series.

Consequently, in conjunction with quantum chemistry calculations, ForMileS contributes to the chemical understanding that F1+ for DGDE must assume a double-bonded structure to achieve a reasonable Gibbs energy level, or a cyclic structure if other factors beyond thermodynamic stability are considered. This constraint arises simply because these are the sole structures that correspond to the molecular formula/exact mass of the fragment.

This illustrates how the confluence of all parameters, particularly exact mass, which is an experimental value, provides theoretical evidence for the mandatory presence of double bonds within the ensemble of structures for F1+, assuming the elemental composition is accurately proposed. Notwithstanding, as previously discussed, all results from ForMileS necessitate meticulous examination within a physical and chemical context, as the current iteration of the program does not incorporate any energy calculation to filter the final results.

## Conclusion

Drawing inspiration from extant molecular graph generator programs and theoretical analyses of MS^2^ dissociation mechanisms, we introduce an innovative open-source Python-based workflow designed for generating molecular graphs within the context of MS^2^. This workflow incorporates the well-established branch-and-bound (B&B) algorithm. Featuring a graphical user interface (GUI), ForMileS leverages parameters pertinent to both precursor and fragment ions to produce sets of fragment ion structures—a distinct focus from numerous programs that primarily emphasize precursor ion elucidation. Through its application to Polypropylene Glycol Octamer (PPG8) and Dipropylene Glycol Dimethyl Ether (DGDE) fragments, coupled with relative energy calculations, we demonstrate that the putative fragment structures are indeed present within the final generated set. Furthermore, the program’s capability to generate coordinate files facilitates its integration with computational chemistry routines, such as DFT and semiempirical calculations. The selection guidelines for the Base Molecular Scaffold (BMS) were also discussed, highlighting its relationship with precursor ions. For DGDE, the program even contributes to ascertaining the chemical significance of the requisite presence of double bonds or cyclic structures for potentially generated structures. Despite its efficacy in reducing computational time and facilitating application, BMS remains a purely hypothetical parameter that critically biases the ultimate results. Additionally, the program exhibits very limited performance compared to already published molecular graph generators and is currently restricted to small molecules, such as C_6_O_3_H_19_, primarily due to the fully combinatorial charge generation step. Future endeavors will prioritize performance optimization and the implementation of additional filters to ensure chemical validation and pruning, leading to more physically and chemically meaningful final results.

## Data Availability

The ForMileS program is open-source, released under GNU GPN v3.0. Source-code, examples, and tutorials presented in this study are openly available in https://github.com/Kuchenbecker/ForMileS, Repository ID 976333496 (repository metadata information). The data underlying this study are openly available in https://beta.mzcloud.org/DataViewer/app#/app mzCloud ID 17,722 (legacy ID 3032) and 5,419 (legacy ID 5474). ThermoFischer Free Account Login is necessary to access its database.

## Abbreviations

MGG: Molecular Graph Generator
CASE: Computer Assisted Structure Elucidation
MS: Mass Spectrometry
MS2: Tandem Mass Spectrometry
PPG8: Polypropylene Glycol Octamer
DGDE: Dipropylene Glycol Dimethyl Ether
SMILES: Simplified Molecular Input Line Entry System
BMS: Basic Molecular Scaffold.
